# Lower limb and feet wound image dataset

**DOI:** 10.1016/j.dib.2026.112730

**Published:** 2026-03-27

**Authors:** Md Darun Nayeem, Md Sakibul Hassan Rifat, Nusrat Jahan Nisita, Md Masudul Islam, Md Saifur Rahman

**Affiliations:** Bangladesh University of Business and Technology, Dhaka, Bangladesh

**Keywords:** Wound dataset, Healthy feet, Lower limb imaging, Segmentation masks, Medical image analysis, Wound classification, Clinical dataset

## Abstract

A comprehensive wound-related image repository was developed to address critical gaps in existing medical imaging resources, particularly the lack of balanced datasets representing both healthy and pathological lower-limb conditions. The collection comprises 5443 images sourced from two complementary streams: real-world clinical wound cases and controlled acquisition of healthy feet images. The wound component includes 2686 expertly annotated images representing eight clinically significant wound types—diabetic, pressure, trauma, venous, surgical, arterial, cellulitis, and miscellaneous categories. These images were gathered across diverse clinical environments between 2015 and 2019 and meticulously annotated by certified wound specialists, ensuring high-quality segmentation masks including peri‑wound regions. The healthy-foot component consists of 2757 images captured from volunteer participants in naturalistic settings using consumer-grade smartphone cameras. Each participant contributed eight multi-angle images under consistent protocols, enabling robust representation of anatomical variability across sex, skin tone, and foot structure. All images were standardized through controlled resizing procedures, while the wound dataset underwent additional mask generation and augmentation strategies to support downstream segmentation and classification tasks. This unified dataset provides a balanced foundation for developing machine learning models capable of distinguishing between normal and pathological foot conditions while supporting advanced tasks such as wound segmentation, severity assessment, and clinical decision support. By integrating healthy and wound images within a single accessible collection, the dataset mitigates class imbalance issues prevalent in existing resources and enables scalable, generalizable deep learning research in wound detection, monitoring, and medical image analysis.

Specifications Table

**Table:** 

Subject	Computer Sciences
Specific subject area	Image dataset for lower limb & feet wound and normal/healthy class for medical image analysis
Type of data	Image, Raw, segmentation mask
Data collection	The dataset was collected through two complementary processes: real-world wound images were gathered from clinical environments and professionally annotated by certified wound specialists, while healthy feet images were captured from volunteer participants in natural classroom settings using smartphone cameras. Each volunteer provided multi-angle images following a consistent protocol, and all wound samples underwent expert mask annotation and quality checks.
Data source location	The healthy feet images were collected at Bangladesh University of Business and Technology (BUBT), Dhaka, Bangladesh, located at 23.8286° N, 90.4250° E. The wound images were sourced from diverse clinical wound-care environments associated with Woundtech specialists across the United States
Data accessibility	Repository name: Mendeley Data Data identification number: 10.17632/hsj38fwnvr.2 Direct URL to data: https://data.mendeley.com/datasets/hsj38fwnvr/2 Instructions for accessing these data: Go to direct repository URL then click to download ZIP file. There are 3 folders: two folders (normal & wound_main) for classification and another folder (wound_mask) is for segmentation purpose.
Related research article	https://ieeexplore.ieee.org/document/10030591

## 1 Value of the Data

• The dataset provides a balanced collection of healthy and wounded lower-limb images, enabling robust development and benchmarking of machine learning models for binary classification, multi-class wound categorization, and semantic segmentation tasks.
• The availability of expert-annotated segmentation masks supports precise segmentation benchmarking, allowing researchers to evaluate and compare deep learning architectures such as U-Net, DeepLab, and transformer-based models under standardized conditions.
• The inclusion of both healthy and pathological samples enables transfer learning, anomaly detection, and contrastive learning frameworks, where healthy images can serve as negative controls to improve generalization in wound detection systems.
• Compared to existing wound datasets, this dataset offers a more balanced representation of healthy and wound classes, along with multi-angle healthy-foot images and peri‑wound segmentation annotations, enhancing its utility for both classification and segmentation research.
• The dataset exhibits high diversity in anatomical structures, wound types, and imaging conditions, improving model robustness across variations in skin tone, lighting, and capture devices—factors often underrepresented in existing datasets.
• The use of consumer-grade smartphone imaging makes the dataset particularly suitable for developing low-cost, deployable AI solutions, including mobile-based wound monitoring systems and telemedicine applications in resource-constrained environments.
• The dataset can further support advanced clinical research tasks such as wound severity grading, progression analysis (when combined with external datasets), and decision-support system development, thereby bridging the gap between experimental AI models and real-world healthcare applications.
• Despite these advantages, users should consider limitations such as the lack of longitudinal data and the geographically localized healthy cohort when interpreting generalization performance.

## 2 Background

Accurate analysis of lower-limb wounds is essential for early diagnosis, treatment planning, and continuous monitoring of chronic wound conditions. Existing wound image repositories, such as the Medetec Wound Database [1] and the AZH foot ulcer dataset introduced by Wang et al [2], have contributed significantly to advancing automated wound segmentation and classification. However, these datasets primarily focus on wounded feet and offer limited diversity in imaging perspectives, ulcer categories, and anatomical variability. Moreover, publicly available datasets rarely include corresponding images of healthy feet, creating a substantial class imbalance that restricts the development of robust machine learning models. Recent studies emphasize the need for large, diverse, and well-annotated wound datasets to improve segmentation accuracy and downstream clinical tasks such as wound area and volume estimation [2,3]. Oota et al [3] highlighted challenges such as heterogeneous wound appearance, varying lighting conditions, and limited annotated data, motivating the creation of more comprehensive resources. To address these gaps, we consolidated a diverse collection of clinical wound images enriched with expert-generated segmentation masks [3] and complemented them with a newly acquired dataset of healthy feet images captured under controlled, real-world settings. This combined dataset provides balanced representation, multi-angle perspectives, and high variability across patient demographics, offering improved support for developing generalizable deep learning models in wound detection, segmentation, and medical decision support.

## 3 Data Description

**Fig. 1:**
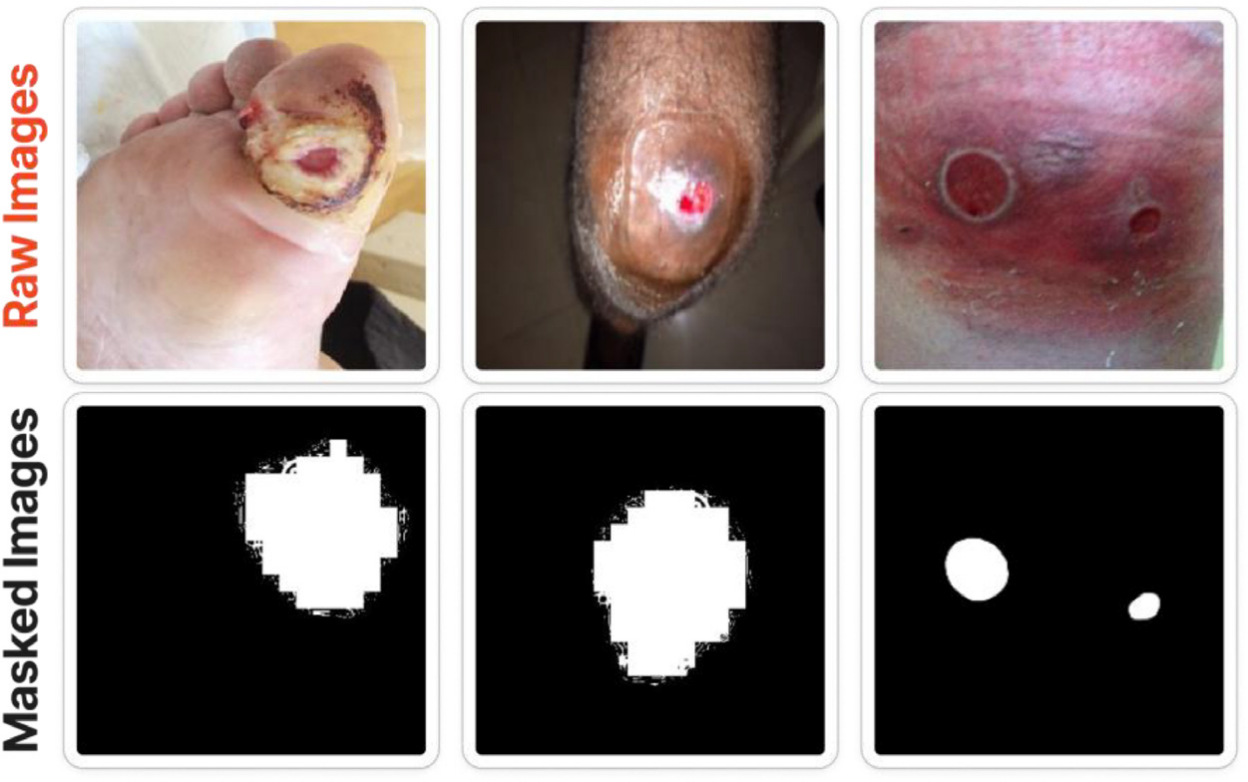
Example of wound raw images and segmented (Binary Masked) images.

The compiled dataset integrates healthy-foot images, clinical wound photographs, and corresponding binary segmentation masks to create a comprehensive resource for lower-limb medical image analysis. A total of 2757 healthy feet images were collected from volunteer participants in real-world classroom environments using consumer-grade smartphone cameras. Images were collected from approximately 345 volunteer participants. Each participant contributed eight multi-angle images, organized into male and female categories to capture anatomical and demographic variability. These images were stored initially in raw JPEG format and later standardized through controlled bulk resizing. The healthy-foot subset is intentionally provided with image-level labels only. Its primary purpose is to support binary classification, anomaly detection, and contrastive representation learning against pathological samples. Unlike wound images, pixel-wise annotation is unnecessary for healthy samples, which serve as negative controls for segmentation tasks and as balanced counterparts for classification benchmarks. Complementing this collection, 2686 clinical wound images were sourced from diverse wound-care settings and represent eight major wound categories, including diabetic, pressure, venous, trauma, surgical, arterial, cellulitis, and other ulcer types. Each wound image underwent expert annotation by certified wound specialists, resulting in high-quality binary segmentation masks that delineate wound and peri‑wound regions. All wound images were resized to 331×331 pixels for uniform benchmarking, whereas their binary masks maintained non-uniform resolutions between 224×224 and 331×331 pixels. Combined with the healthy-foot images, the 2686 annotated wound samples constitute a diverse dataset supporting classification, segmentation, and clinical decision-support studies. The segmentation masks are provided as binary images (PNG format), where pixel values represent two classes: wound (foreground) and background (non-wound region). In the mask images, wound and peri‑wound regions are jointly labeled as the foreground class to ensure comprehensive coverage of clinically relevant tissue. Background pixels correspond to healthy surrounding skin and non-relevant regions. This binary labeling scheme simplifies integration with standard semantic segmentation models. The dataset is organized into three primary directories to support different machine learning tasks. The “normal” folder contains all healthy-foot images, which are intended for classification and anomaly detection tasks. The “wound_main” folder includes all wound images categorized into eight clinically relevant classes, while the “wound_mask” folder contains the corresponding binary segmentation masks for each wound image. Each wound image in the “wound_main” directory has a matching mask file with identical file naming to ensure correct pairing. This structure enables flexible usage of the dataset for both classification and segmentation workflows. For reproducible segmentation experiments, we recommend the following preprocessing pipeline: (i) resize both image and mask to a unified resolution (256×256 or 331×331), (ii) normalize RGB channels to [0,1], (iii) optionally apply histogram equalization, and (iv) perform synchronized augmentations such as rotation, flipping, and scaling. These steps ensure stable training across deep learning architectures. This integrated dataset addresses existing limitations in wound-related image resources by providing balanced representations of normal and pathological conditions essential for developing robust and generalizable AI systems. A detailed breakdown of class distributions is provided in Table 1, while representative examples of raw wound images and their corresponding segmentation masks are illustrated in Fig. 1.

**Table 1:** Summary of wound image dataset and its classes.

Class	Types	Total Images	Wound Area	Example Images
Wound	Diabetic	441	9%	
	Pressure	636	12.5%	
	Trauma	368	10.7%	
	Venous	690	13.3%	
	Surgical	268	12.5%	
	Arterial	99	8.7%	
	Cellulitis	113	13%	
	Other	71	18.9%	
	TOTAL	2686	-	
Normal	Male	1981		
	Female	776		
Total Images		5443

## 4 Experimental Design, Materials and Methods

The dataset was developed through a systematic multi-stage procedure integrating healthy-foot images with clinically sourced wound images and corresponding binary segmentation masks. For the healthy-foot component, data were collected at the Bangladesh University of Business and Technology (BUBT), Dhaka, in natural classroom environments under ambient lighting. Volunteer participants were instructed to position each foot for four distinct angles per leg, resulting in eight images per person. Consumer-grade smartphones (Realme Narzo 20 Pro, Realme Narzo 50, Vivo Iqoo Neo 9) were used for image acquisition to replicate realistic low-cost imaging conditions. Images were stored in raw JPG format and subsequently resized in bulk to ensure uniform spatial resolution across the dataset. During the resizing process, images were scaled using standard interpolation methods (bilinear interpolation for images and nearest-neighbor interpolation for segmentation masks) to preserve spatial consistency. No additional color filtering or enhancement was applied to the released dataset to maintain the original visual characteristics. However, users are encouraged to apply normalization techniques (e.g., scaling pixel values to [0,1] or standardization) during model training depending on their experimental setup.

**Fig. 2:**
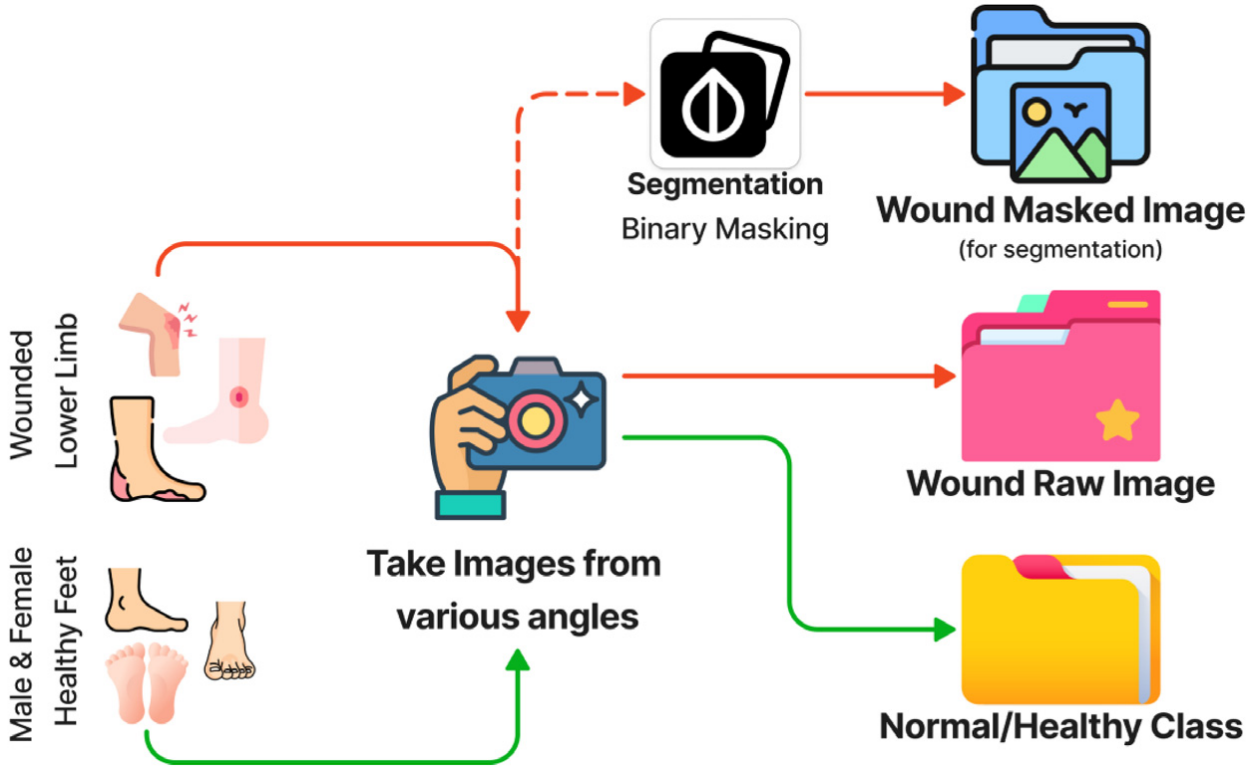
Data collection and pre-processing workflow.

In parallel, clinical wound images were obtained from diverse wound-care settings between 2015 and 2019. An initial collection of approximately 3000 images representing eight wound categories—diabetic, pressure, venous, trauma, surgical, arterial, cellulitis, and miscellaneous—was curated for annotation. Annotation was performed using the Django-labeler tool by three qualified wound specialists, who segmented both the wound core and peri‑wound tissue. Annotation quality was evaluated using the Dice Similarity Coefficient (DSC) between repeated segmentations, yielding an average intra-annotator agreement of approximately 0.95. After removing low-quality or ambiguous samples, 2686 wound images with corresponding binary segmentation masks were finalized. All wound images were standardized to 331×331 pixels for consistent model training and benchmarking, while the corresponding binary masks were resized to non-uniform resolutions ranging from 224×224 to 331×331 pixels. This dataset in stored in publicly available repository in Mendeley Data [4]. Fig. 2 illustrates the detail process of our dataset collection in following steps:

Step 1: Gather two streams—clinical wounded lower-limb photos and volunteer healthy-feet photos.
Step 2: Take images from multiple angles using smartphone cameras under indoor lighting.
Step 3: Store raw JPEGs and run a quality check to remove blurred or duplicate shots.
Step 4: Sort images into two folders: Wound Raw Images and Normal/Healthy Class for classification tasks.
Step 5: For each wound image, perform expert segmentation of wound and peri‑wound tissue in an annotation tool.
Step 6: Export the segmentation as binary masks.
Step 7: Pair each wound image with its mask to create Wound Masked Images for segmentation experiments.

The technical configuration of the smartphone cameras used in this dataset is summarized in Table 2.

**Table 2:** Smartphone camera settings used for image acquisition.

Specification	Realme Narzo 20 Pro	Realme Narzo 50	Vivo Iqoo Neo 9
Rear Camera (Primary)	48 MP, f/1.8, PDAF	50 MP, f/1.8, PDAF	50 MP, f/1.9, OIS
Additional Lenses	8 MP ultrawide, 2 MP macro, 2 MP depth	2 MP depth sensor	8 MP ultrawide
Resolution Used	4096×3072 pixels (JPEG)	4096×3072 pixels (JPEG)	4080×3000 pixels (JPEG)
Lighting	Natural indoor classroom lighting	Natural indoor classroom lighting	Natural indoor classroom lighting
Storage	Internal device memory	Internal device memory	Internal device memory

**Fig. 3:**
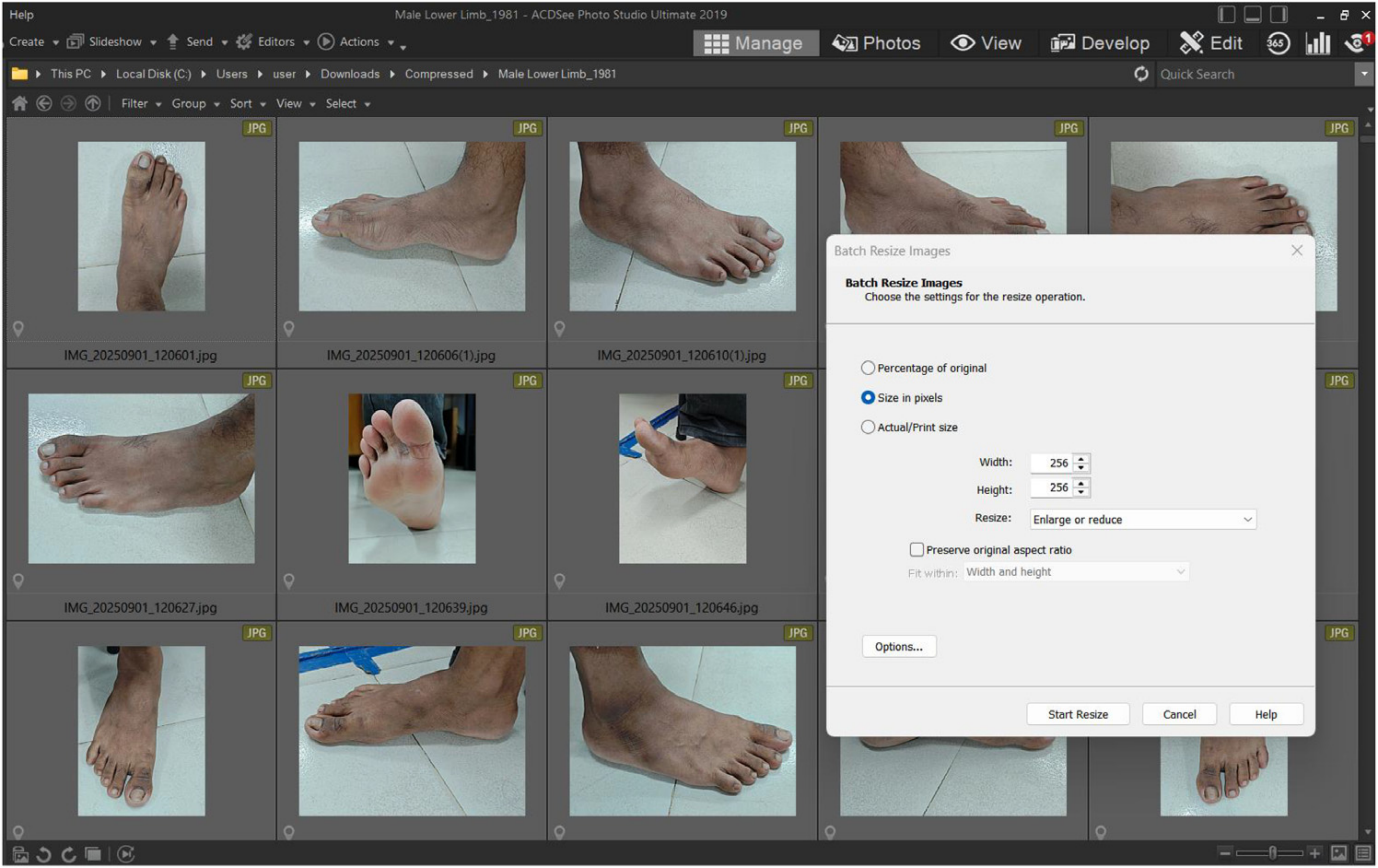
Image pre-processing tools for bulk image resizing & renaming.

Before publish the raw images in data repository a bulk resize is performed in our dataset using an image processing tools called “ACDSee Photo Studio Ultimate” which is shown in Fig. 3.

## Limitations

While this dataset provides a valuable resource for wound analysis and lower-limb image research, several limitations should be acknowledged. First, the clinical wound images originate from diverse care environments without uniform control over lighting, imaging distance, or camera specifications, which may introduce variability that can affect model performance. Additionally, although expert-annotated masks ensure high-quality segmentation labels, the non-uniform resolutions of the binary masks—resulting from differences in annotation boundaries and pre-processing—may pose challenges for certain deep learning architectures requiring strictly aligned input dimensions. The dataset also does not include longitudinal wound progression images, preventing analysis of temporal healing patterns. Furthermore, healthy-foot images were collected from a single geographic region, which may limit demographic diversity in skin tones and anatomical characteristics. Lastly, the dataset is intended solely for research and should not be used for clinical decision-making without external validation in real-world healthcare settings.

## Ethics Statement

This study integrates data from two sources: a publicly available wound image dataset and a newly collected healthy-feet image dataset. Healthy Feet Images (Primary Data): This component of the study involved voluntary participation by individuals who contributed non-identifiable lower limb and feet images. No personal, sensitive, or health-related information was collected, and all data were fully anonymized prior to analysis. Participants were informed about the purpose and procedures of the study and provided written informed consent prior to image acquisition. The data collection was non-invasive and carried out in accordance with the Declaration of Helsinki. In accordance with the institutional guidelines of Bangladesh University of Business and Technology, formal ethical approval was not required for this anonymous, non-clinical data collection as it did not involve identifiable human subjects or sensitive data. Wound Images (Secondary Data): The clinical wound images and segmentation masks were obtained from a publicly available, open-access repository (https://huggingface.co/datasets/subbareddyoota/wseg_dataset) which is under MIT license, which permits reuse, redistribution, and derivative works with appropriate attribution. These data were originally collected and shared by previous researchers in accordance with their institutional ethical standards. As secondary data, no further ethical approval or participant consent was required for their reuse in this study.

## CRediT Author Statement

**Md. Darun Nayeem:** Investigation, Software, Validation, Formal Analysis, Methodology, Resources, Data Curation. **Md Sakibul Hassan Rifat:** Data Curation. Nusrat Jahan Nisita: Data Curation. **Md. Masudul Islam:** Project administration, Conceptualization, Methodology, Formal analysis, Resources, Writing - Original Draft, Visualization. **Md. Saifur Rahman:** Supervision.

## Data Availability

Mendeley DataLower Limb and Feet Wound Image Dataset for Medical Analysis (Original data).
